# Broader Neutralizing Antibodies against H5N1 Viruses Using Prime-Boost Immunization of Hyperglycosylated Hemagglutinin DNA and Virus-Like Particles

**DOI:** 10.1371/journal.pone.0039075

**Published:** 2012-06-13

**Authors:** Shih-Chang Lin, Yu-Fen Lin, Pele Chong, Suh-Chin Wu

**Affiliations:** 1 Institute of Biotechnology, National Tsing Hua University, Hisnchu, Taiwan; 2 National Institute of Infectious Diseases and Vaccinology, National Health Research Institutes, Zhunan, Miaoli County, Taiwan; Johns Hopkins University – Bloomberg School of Public Health, United States of America

## Abstract

**Background:**

Highly pathogenic avian influenza (HPAI) H5N1 viruses and their transmission capability from birds to humans have raised global concerns about a potential human pandemic. The inherent nature of antigenic changes in influenza viruses has not been sufficiently taken into account in immunogen designs for broadly protective HPAI H5N1 vaccines.

**Methods:**

We designed a hyperglycosylated HA vaccine using N-linked glycan masking on highly variable sequences in the HA1 globular head. Immunization of these hyperglycosylated HA DNA vaccines followed by a flagellin-containing virus-like particle booster in mice was conducted to evaluate neutralizing antibody responses against various clades of HPAI H5N1 viruses.

**Results:**

We introduced nine N-X-S/T motifs in five HA1 regions: 83NNT, 86NNT, 94NFT, 127NSS, 138NRT, 156NTT, 161NRS, 182NDT, and 252NAT according to sequence alignment analyses from 163 HPAI H5N1 human isolates. Although no significant differences of anti-HA total IgG titers were found with these hyperglycosyalted HA compared to the wild-type control, the 83NNT and 127NSS mutants elicited significantly potent cross-clade neutralizing antibodies against HPAI H5N1 viruses.

**Conclusions:**

This finding may have value in terms of novel immunogen design for developing cross-protective H5N1 vaccines.

## Introduction

Highly pathogenic avian influenza (HPAI) H5N1 viruses and their transmission capability from birds to humans have raised global concerns about a potential human pandemic, with new H5N1strains emerging and evolving. The World Health Organization (WHO) has classified recently isolated H5N1 viruses into 10 clades or sublineages, based on phylogenetic analysis of viral hemagglutinin (HA) sequences [1]. With the ongoing threat of an influenza pandemic arising from avian reservoirs, the development of broadly protective vaccines is particularly important. To date, such vaccines have been achieved such as using novel adjuvant formulations [2].

However, the inherent nature of antigenic changes in influenza viruses has not been sufficiently taken into account in immunogen designs for broadly protective H5N1 vaccines. One approach is to refocus antibody responses by designing immunogens that can preserve overall immunogen structure, but selectively mutate “undesired” antigenic sites that are highly variable (i.e., mutants that evade protective immune responses), immunosuppressive (i.e., downregulate immune responses to infections), or cross-reactive (i.e., immune responses induce reactions to proteins resembling immunogen) [3]–[9]. By refocusing antibody responses, the immunogen design has been applied to HIV-1 vaccines- that is, hyperglycosylated HIV-1 gp120 immunogens have been used, with undesired epitopes masked by the selective incorporation of N-linked glycans [4], [6], [10]–[12]. This glycan-masking strategy has also been used in the design of vaccines aimed at enhancing antibody responses to a broad range of H3N2 intertypic viruses [13]. However, to date there are no reports for glycan-masking immunogens for H5N1 vaccines.

DNA vaccines offer advantages in terms of genetic antigen design, manufacturing time, stability in the absence of cold chains and immunogenicity elicited by T cells via endogenerous antigen processing pathways [14]. The problem of low DNA immunogenicity in large animals and humans has been overcome through the use of novel delivery systems such as gene-guns and electroporation [14]. Furthermore, DNA vaccine-elicited immune responses can be augmented by heterologous prime-boost immunization regimens, in which booster doses use a different vaccine format containing identical or similar antigens. DNA vaccine prime-boost immunization strategies have been described for inactivated influenza viruses [15], [16], live-attenuated influenza viruses [17], recombinant adenoviruses [18], virus-like particles (VLPs) [19], [20] and recombinant subunit proteins in adjuvants [21]–[25]. Humans receiving H5 DNA vaccine priming followed by a booster with an inactivated H5N1 vaccine were found to enhance the protective antibody responses, and in some cases induce hemagglutinin stem-specific neutralizing antibodies [16].

**Figure 1:**
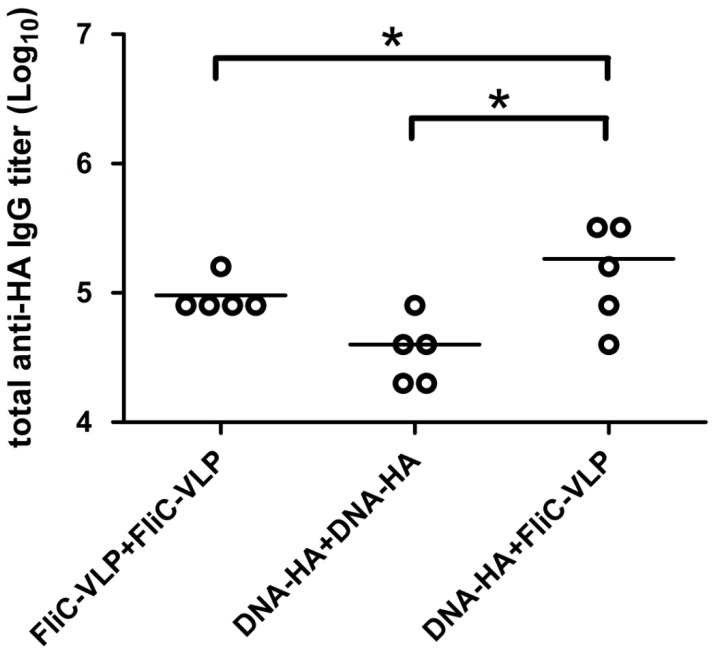
Total anti-HA IgG titers elicited by DNA-HA and FliC-VLPs. BALB/c mice were immunized using a prime boost regimen over a three-week interval as shown: (i) FliC-VLP+FliC-VLP, (ii) DNA-HA+DNA-HA, or (iii) DNA-HA+FliC-VLP. Sera were collected two weeks following the booster immunization. Results indicate significantly higher HA-specific IgG titers from DNA-HA vaccine vector priming followed by FliC-VLP boosting. Shown are individual titers (points) and geometric means (lines) for each group. Asterisks (*) indicate statistical significance at p<0.05.

**Figure 2:**
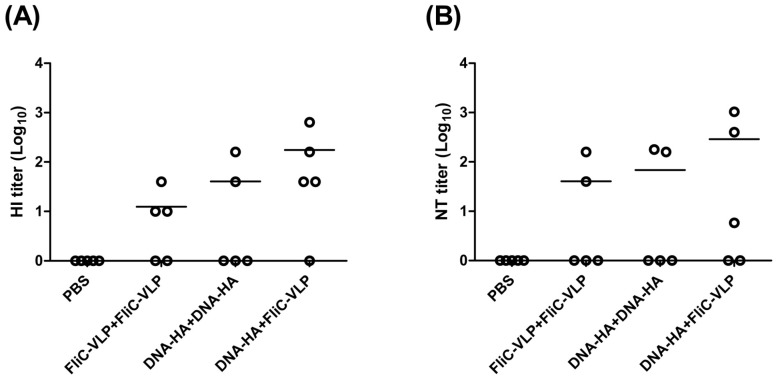
HI and NT titers against the NIBRG-14 virus. Neutralizing activities in sera collected from immunized mice were determined by measuring (A) HI and (B) NT titers against the NIBRG-14 (clade 1) H5N1 influenza virus. Results showed that the DNA-HA vector priming/FliC-VLP booster regiment elicited the highest magnitude of neutralizing antibodies. For calculation purposes, undetectable titers were scored as 1. Individual titers (points) and geometric means (lines) are given for each group.

For this study we designed a hyperglycosylated HA vaccine using N-linked glycan masking on highly variable sequences in the HA1 globular head. Priming with hyperglycosylated HA DNA vaccine followed by a booster of flagellin-containing influenza virus-like particles (FliC-VLPs) in mice. FliC is a Toll-like receptor 5 (TLR-5) ligand and has been widely used for vaccine design, for its ability to induce the innate immune effectors, like cytokine and nitric oxide, e.g. induction of macrophage nitric oxide production [26] and activation of interleukin-1 receptor-associated kinase [27], thereby stimulating the activation of adaptive immune response. We previously reported that the influenza VLP can be fabricated by M2 fusion with FliC to improve and broaden the elicited neutralizing antibodies against homologous and heterologous HPAI H5N1 viruses [28]. We hope these findings have value in terms of novel immunogen design for developing cross-protective H5N1 vaccines.

**Figure 3:**
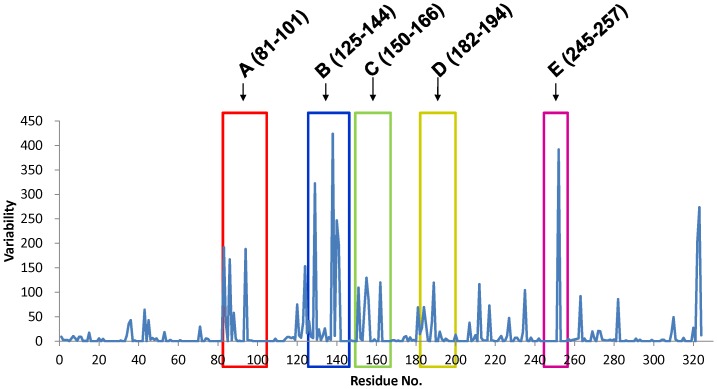
HA sequence alignment. Results from analyses of amino acid variation in the HA1 of 163 avian influenza virus strains reveal five regions with the highest variability scores (0 to 4 based on Vector NTI Tables). HA1 subunit regions A (HA81–101), B (HA125–144), C (HA150–166), D (HA182–204), and E (HA245–257) were found to have higher scores.

**Figure 4:**
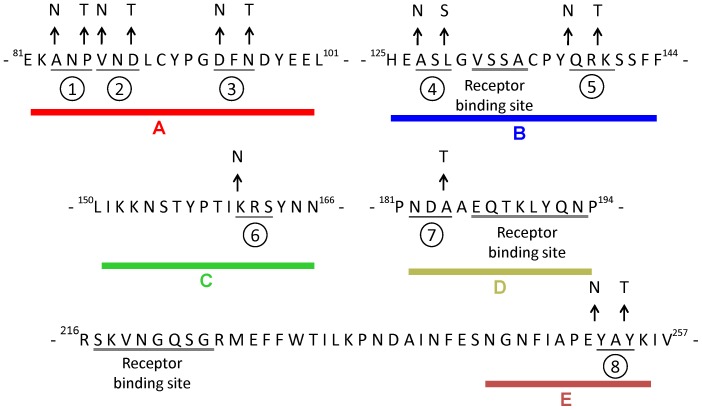
Hyperglycosylated DNA design. Site-directed mutagenesis in five HA1 subunit regions (A, B, C, D, E) was performed following the addition of N-linked glycosylation sites. Nine N-X-S/T motifs were created in the five regions: (1) 83NNT, (2) 86NNT, (3) 94NFT, (4) 127NSS, (5) 138NRT, (6) 156NTT, (7) 161NRS, (8) 182NDT, (9) 252NAT. Triplet amino acids are underlined. Arrows point away from wild-type sequences to amino acid changes resulting in N-linked glycosylation sequences.

## Materials and Methods

### DNA-HA vaccine vector construction

Complimentary DNA (cDNA) from the HA gene of the A/Thailand/1(KAN-1)/2004/H5N1 influenza virus (clade 1) was generously provided by Prasert Auewarakul of Siriraj Hospital, Thailand. A full-length HA sequence was inserted into a pcDNA™3.1(+) vector (Invitrogen) via a KpnI/NotI cut site. The HA-containing plasmid was transfected into 293 cells using Turbofect reagent (Fermentas). At 48 h post-transfection, cell lysates were collected by centrifugation at 5000 rpm for 10 minutes, and HA expression was analyzed by Western blotting using anti-H5N1 HA antibodies (ab21297; Abcam).

**Figure 5:**
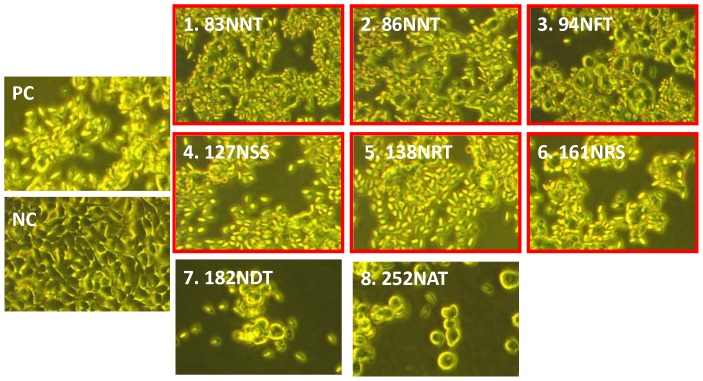
Hemadsorption assay. RBCs were added to 293 cells transfected with mutated DNA-HA vectors to cover cell monolayer. Results for RBC adsorption indicate that cells transfected with the 83NNT, 86NNT, 94NFT, 127NSS, 138NRT and 161NRS vectors retained their hemadsorption function, while cells transfected with the 156NTT, 183NDT and 252NAT vectors did not. Cells transfected with a wild-type DNA-HA vector were used as a positive control (PC); cells tranfected with an empty vector were used as a negative control (NC).

**Figure 6:**
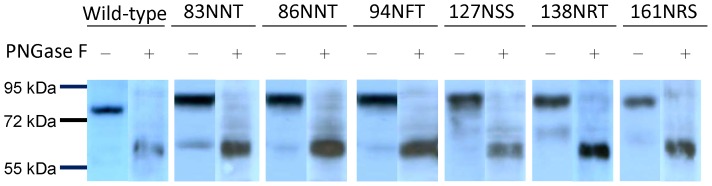
Hyperglycosylated HA characterization. Cell lysates from 293 cells transfected with hyperglycosylated DNA-HA vectors were treated with PNGase F to determine changes in HA molecular weights. HA mutant proteins expressed by the DNA-HA vectors with added N-linked glycans (83NNT, 86NNT, 94NFT, 127NSS, 138NRT and 161NRS) all had increased molecular weights that decreased following PNGase F treatment.

### HA glycosylation patterns and trypsin treatment

To characterize HA glycosylation patterns, 293 cells were harvested following transfection with DNA-HA vectors for 48 h. Cell lysates were treated with Endo H or PNGase F for 2 h at 37°C, and HA glycosylation patterns were determined by Western blotting. For trypsin treatment, cell lysates were incubated with trypsin for 30 min on ice, and HA0 cleavage into HA1 and HA2 was confirmed by Western blotting.

### FliC-VLP preparation

FliC-VLPs were prepared as described previously [26] Briefly, HA and M1 were cloned into one pFastBac™ dual vector (Invitrogen). NA and FliC-M2 (expressing FliC-M2 fusion proteins) were cloned into the other pFastBac™ dual vector. Two recombinant baculoviruses were obtained to infect Sf9 cells (Invitrogen) for FliC-VLPs production. Cell culture supernatants were harvested at 72 h post-infection and concentrated by filtration using a 500 kDa filter membrane. Concentrates were loaded onto 0–60% sucrose gradients and centrifuged for 4 h at 33,000 rpm. Particles were measured by Western blotting using the following antibodies: anti-H5N1 HA antibodies (ab21297; Abcam), anti-NA antibodies (ab70759; Abcam), anti-M1 antibodies (ab25918; Abcam), and anti-M2 antibodies (NB100–2073; Novus). Particles were also observed by transmission electron microscopy (TEM) as described previously [26].

**Figure 7:**
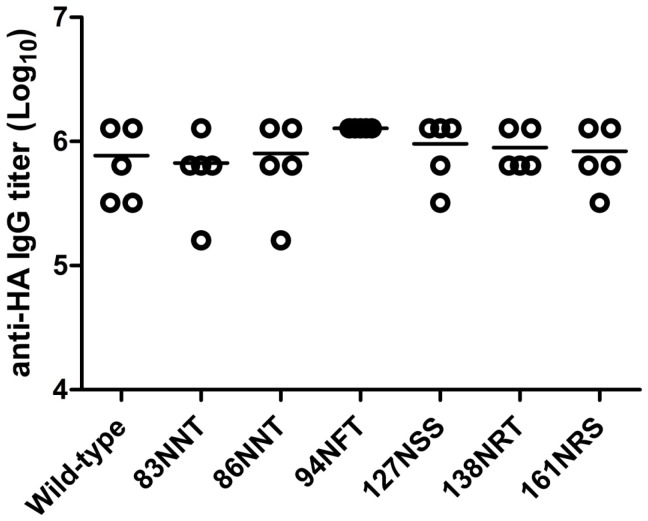
Total anti-HA IgG titers elicited by hyperglycosylated DNA vaccine priming followed by FliC-VLP boosting. Mice were immunized with each immunorefocusing HA vector twice followed with a third boosting dose with FliC-VLPs on a three-week interval. Sera were collected at 2 weeks after final immunization, and determined the anti-HA IgG titers by ELISA. The results showed that no significant differences of the HA-specific total IgG titers of all the immunized groups with the hyperglycosyalted HA DNA vaccines compared to the wild-type control. Individual titers (points) and geometric means (lines) are given for each group.

**Figure 8:**
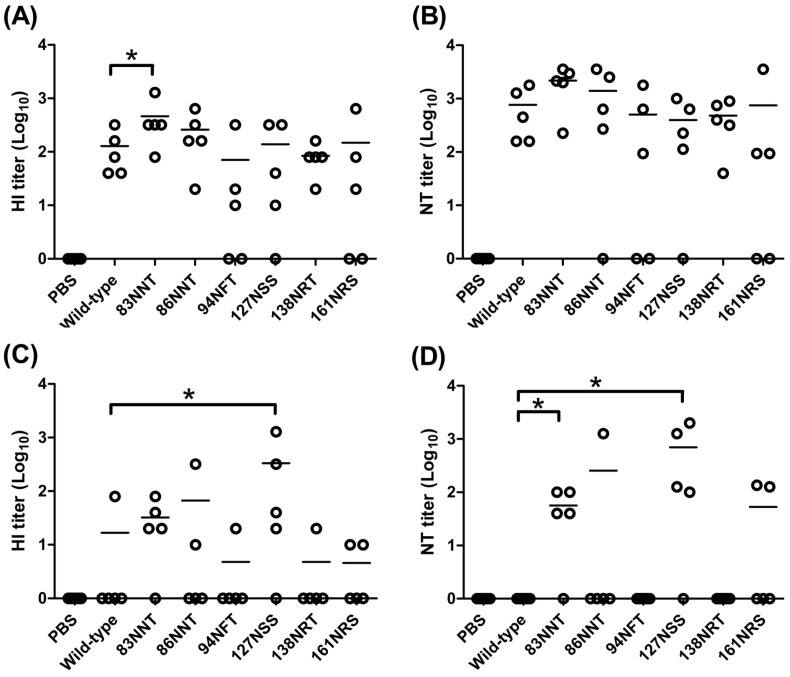
HI and NT titers against the NIBRG-14 and A/whooper swan/Mongolia/244/2005 viruses. Neutralizing activity in sera collected from immunized mice was determined by measuring (A) HI and (B) NT titers against the A/Vietnam/1194/2004 (clade 1) H5N1 virus, and (C) HI and (D) NT titers against the A/Whooper Swan/Mongolia/244/2005 (clade 2.2) H5N1 virus. For calculation purposes, an undetectable level was scored as titer = 1. Individual titers (points) and geometric means (lines) are given for each group. Asterisks indicate statistical significance at p<0.05.

### Hyperglycosylated HA preparation

Mutations were introduced into the HA gene by site-directed mutagenesis, with plasmids encoding a wild-type HA gene (i.e. A/Thailand/1(KAN-1)/2004) used as templates. PCR reactions (50 µL) were performed using 100 ng templates, 2 mM primer pair, 200 mM dNTPs, and 2U of DNA polymerase. PCR products were purified and treated with DpnI for 2 h at 37°C. DpnI treated products were transformed into TOP10 competent cells prior to the mutated plasmids.

### Hemadsorption assay

293 cells (BCRC, FIRDI, Taiwan) were transfected with wild-type and mutated DNA-HA vectors; cells were harvested 72 h post infection. After washing with phosphate-buffered saline (PBS), 0.5% turkey red blood cells (RBCs) were added to cover the cell monolayer, followed by incubation for 30 min at room temperature. RBC adsorption was observed after two rinses with PBS.

**Figure 9:**
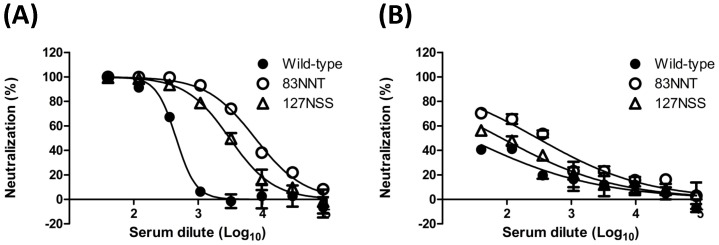
Neutralization against H5pp of A/Anhui/1/2005 and A/Hubei/1/2010 strains. Dose-dependent neutralization curves were plotted against (A) A/Anhui/1/2005 and (B) A/Hubei/1/2010 H5N1 influenza strains. Data indicate that the 83NNT and 127NSS HA mutants elicited higher neutralizing antibodies than the wild-type HA.

### Mouse immunization

A homologous/heterologous prime-boost regimen was used to immunize female BALB/c mice (6–8 weeks old) with 50 µg of DNA and 30 µg of purified FliC-VLPs mixed with Alum adjuvant in PBS. Immunizations were performed by intramuscular injection during weeks 0 and 3 for the two-dose regimens, and weeks 0, 3, and 5 for three-dose regimens. Blood was collected and serum isolated 14 d after the final booster dose. Serum samples were inactivated by incubation at 56°C for 30 min and stored at −20°C. All experiments were conducted in accordance with guidelines established by the Laboratory Animal Center of National Tsing Hua University (NTHU). Animal use protocols were reviewed and approved by the NTHU Institutional Animal Care and Use Committee (approval no. 09733).

### Enzyme-linked immunosorbent (ELISA) assay

Recombinant HA proteins were obtained from baculovirus-infected Sf9 cells and ELISA assays were performed as described previously [29]. Briefly, 2 µg/mL of purified HA proteins were coated onto 96 well plates and blocked with BSA. Serial dilutions of each serum sample were incubated in the plates for 1 h at 37°C and removed by 3 more washes with PBST. Goat anti-mouse IgG conjugated HRP (Bethyl Laboratories, Inc.) was incubated in the plates for 1 h at 37°C followed by 3 more washes with PBST. Following reaction with TMB substrate, stop solution, plates were read at 450 nm absorbance. End-point titers were determined as reciprocals of final dilutions, giving an optical of two-fold absorbance of negative control.

### Hemagglutination inhibition (HI) and neutralization (NT) assays

HI and NT assays were performed as described previously [29], [30]. For the HI assay, serum samples (two-fold dilutions starting with an initial dilution of 1∶10) were incubated with four HA units of influenza strain. Turkey RBCs were added and agglutination inhibition was scored. Serum titers were expressed as the reciprocals of the highest dilutions showing complete HA inhibition. For the NT assay, 200 TCID_50_ of virus per well were incubated with two-fold-diluted mice sera (starting dilution 1∶40). Virus and serum mixtures were transferred to MDCK cell (BCRC, FIRDI, Taiwan) monolayers and incubated for 4 d at 37°C. Neutralizing titers were defined as the reciprocals of highest serum dilutions at which H5N1 virus infectivity was neutralized in 50% of the wells. Infectivity was determined by the presence of cytopathy on day 4; titers were calculated using the Reed-Muench method.

### H5 pseudotyped particle (H5pp) neutralization assay

Influenza lentiviral pseudotyped viruses were generated as described previously [29]. Briefly, 293 cells were co-transfected with pNL Luc E^−^ R^−^ and pcDNA3.1(+) expressing HA from A/Hubei/1/2010 (clade 2.3.2) and A/Anhui/1/2005 (clade 2.3.4) strains. *Vibrio cholerae* neuraminidase (6.2 mU/mL; Sigma) was added 24 h post-transfection to release particles from cells. Culture supernatants were collected and concentrated 48 h post-transfection. H5pp titers were determined by p24 ELISA (Clontech). Neutralizing antibodies were quantified as reduced luciferase expression level following H5pp transduction in MDCK cells. MDCK cells (10,000 cells/well) were seeded in 96-well plates in 100 µL DMEM. The following day, H5pp (∼10^5^RLU) was incubated with a three-fold serial dilution of anti-sera for 1 hr at 37°C in 110 µL DMEM. Next, 100 µL of the resulting mixture were transferred to cells. Fresh medium was added again after 24 h; luciferase assays were performed 48 hr later via the direct addition of neolite luciferase substrate (PerkinElmer). Neutralization titers and corresponding standard deviations were determined from neutralization curves using the ID50 program (http://www.ncbi.nlm.nih.gov/CBBresearch/Spouge/html_ncbi/html/index/software.html) developed by John Spouge of the National Center for Biotechnology Information, National Library of Medicine, US National Institutes of Health.

### Statistic analysis

All results were analyzed using two-tailed Student's *t* tests, with statistical significance indicated at *p*<0.05.

## Results

### Prime-boost immunization regimens

To investigate the combinatory use of DNA-HA vaccine vector and influenza VLP for prime-boost immunization studies, we immunized BALB/c mice intramuscularly with two doses within a three-week interval, following the prime-boost regiments (i) PBS+PBS, (ii) FliC-VLP+FliC-VLP, (iii) DNA-HA+DNA-HA, (iv) DNA-HA+FliC-VLP. FliC-VLPs were obtained from bacuclovirus coexpression of influenza HA, NA, M1 and FliC-M2 fusion proteins since the FliC-VLPs have been shown to be more immunogenic than influenza VLPs as we previously reported [28]. Groups of BALB/c mice were immunized with each specified prime-boost regimen and their serum samples were collected two weeks following the second dose. The results show that the HA-specific total IgG titer resulting from DNA-HA vaccine vector priming followed by FliC-VLP boosting was significantly higher than from two-dose immunization regimen using a DNA-HA vector and FliC-VLPs (Fig. 1). According to neutralizing activities (as indicated by measuring HI and NT titers against the NIBRG-14 [clade 1] vaccine virus), the DNA-HA vector priming/FliC-VLP boosting regiment elicited the highest levels of neutralizing antibodies (Fig. 2A–B). To further improve the immunization protocols, DNA-HA vector was used for immunization for two doses to enhance the DNA vaccine-elicited immunity, then followed with a third booster dose of FliC-VLPs. The three-dose immunization regimens (DNA-HA+DNA-HA+FliC-VLP) were chosen for latter experiments.

### Hyperglycosylated HA DNA design based on H5N1 human isolate amino acid sequences

To design the hyperglycosyalted HA DNA vaccines, we performed sequence alignment analyses from 163 HPAI H5N1 human isolates, using sequences retrieved from the NCBI Database. Amino acid differences were scored as 4, different amino acid); 2, weakly similar amino acid; 1, strongly similar amino acid; and 0, identical amino acid, as characterized by Vector NTI tables. Using the alignment plot shown in Fig. 3, we identified five regions that had higher scores, including regions A (HA81–101), B (HA125–144), C (HA150–166), D (HA182–204), and E (HA245–257). To design antibody-refocused immunogens, we performed site-directed mutagenesis in each of the five regions, with mutations that supported the addition of an N-X-S/T motif (for the N-linked glycosylation site) but avoid receptor binding sites (i.e. the B and D regions) [31], [32]. Accordingly, we intrduced nine N-X-S/T motifs in five HA1 regions: 83NNT, 86NNT, 94NFT, 127NSS, 138NRT, 156NTT, 161NRS, 182NDT, and 252NAT (Fig. 4).

### Characterization of hyperglycosylated HA

Refocused hyperglycosylated HA genes containing these eight specified N-linked glycosylation sites were cloned into the DNA-HA vaccine vector. Only the 83NNT, 86NNT, 94NFT, 127NSS, 138NRT and 161NRS mutant DNA-HA vectors retained the hemagglutination property for turkey RBCs following transfection into 293 cells (Fig. 5). These six HA mutant genes (83NNT, 86NNT, 94NFT, 127NSS, 138NRT and 161NRS) were further confirmed for the introduction of N-linked glycans in HA antigens, as illustrated by increased molecular weights and decreases to the same molecular weight following treatment with PNGase F (Fig. 6).

### Antibodies elicited by hyperglycosylated HA DNA vaccines

To investigate antibody responses elicited by the six hyperglycosylated HA mutants, over three weeks we immunized mice with two doses of DNA-HA vector, and followed with a third booster dose with FliC-VLPs. The three-dose immunization strategy (DNA-HA+DNA-HA+FliC-VLP) was applied to improve the DNA vaccine-elicited immune responses by heterologous prime-boost immunization. Our results indicate no significant differences of anti-HA total IgG titers across the immunized groups, with hyperglycosyalted HA DNA vaccines compared to the wild-type control (Fig. 7). However, for the elicitation of functional antibodies, the 83NNT HA mutant was found to have a higher HI titer (p<0.05) (Fig. 8A) but only slightly higher but not statistically significant NT titer (Fig. 8B) against the NIBRG-14 vaccine virus, a vaccine strain virus derived from a reassortant H5N1 vaccine strain containing modified HA and NA from the A/Vietnam/1194/2004 H5N1 virus (clade 1 strain). We also measured HI and NT titers in these sera against the A/Whooper Swan/Mongolia/244/2005 H5N1 virus (clade 2.2 strain). The data (presented as cross-clade functional antibodies) indicate that the 127NSS HA mutant elicited a higher HI titer (p<0.05) (Fig. 8C) and the 83NNT and 127NSS HA mutants elicited higher NT titers (p<0.05) (Fig. 8D). To further confirm the cross-clade neutralizing antibodies, H5pp containing the HA gene of A/Anhui/1/2005 (clade 2.3.4) or A/Hubei/1/2010 (clade2.3.2) was reacted with the serum samples from wild-type, 83NNT, and 127NSS HA immunizations. Dose dose-dependent neutralization curves were all shown for H5pp Anhui (Fig. 9A) and H5pp Hubei (Fig. 9B), showing the cross-clade neutralizing antibodies elicited by the 83NNT HA followed by 127NSS HA immunizations were significantly higher compared to wild-type HA immunization. Combined, the data show that the 83NNT and 127NSS mutants elicit potent cross-clade neutralizing antibodies against HPAI H5N1 viruses.

## Discussion

As H5N1 viruses evolve and diverge into distinct antigenic clades and subclades, the growing challenge is to develop vaccines that can provide broad protective immunity. The WHO has recently recommended 8 H5N1 virus clades/subclades (1, 2, 2.1, 2.2.1, 2.2.3, 2.3.4, 4, 7) as candidates for vaccine preparation [33]. In this study we designed hyperglycosylated HA and used the hyperglycosylated HA DNA as a prime-boost immunization regimen with FliC-VLPs. Immunofocusing antibody responses by priming with the hyperglycosylated HA DNA vaccine plus additional glycan masking at 83-NNT or 127-NNS in the HA1 globular head induced more potent cross-clade neutralizing antibodies against HPAI H5N1 viruses.

A prime-boost immunization regimen represents a straightforward strategy for augmenting immune responses elicited by DNA vaccination. Our results demonstrate that priming with a DNA-HA vaccine followed by a booster of FliC-VLPs induced higher titers of total IgG and functional HI and NT antibodies compared to an immunization regimen of two doses of either DNA or FliC-VLPs along (Fig. 2A–B). Notably, serum samples in the FliC-VLP+FliC-VLP group showed all high binding titers of (10^5^ IgG titers in Fig. 1) but only few individual samples showed HI and NT titers (Fig. 2). The immunizations using influenza VLPs or FliC-VLPs required three doses under our experimental conditions to give all high HI and NT titers where the binding titers reached to approximately 10^6^ IgG titers (data not shown). The heterologous prime-boost regimen consisting of DNA and HIV-gag VLPs have previously been reported as eliciting higher neutralizing antibodies and greater protective immunity against H5N1 and other seasonal influenza viruses [19], [20]. Our results are in agreement with these findings in that the highest levels of neutralizing antibody titers were elicited by DNA-VLP heterologous prime-boost immunization regardless of which influenza FliC and VLP types were used.

Glycan masking on the immunodominant epitopes of surface immunogens can elicit more broadly neutralizing antibodies if immunodominant sites overlap with the sequence variable regions of antigenic sites. We therefore hypothesized that hyperglycosylated HA with extra glycan masking on the HA1 variable regions (A–E in Fig. 3) might boost broadly neutralizing antibodies against HPAI H5N1 viruses. Our results indicate that hyperglycosyalted HA DNA vaccines containing 83NNT mutation in the HA1 globular head resulted in the elicitation of a higher HI titer against the homologous clade virus (A/Vietnam/1194/2004, clade 1) (Fig. 8A–B). Furthermore, hyperglycosyalted HA DNA vaccines containing 83NNT or 127NSS mutation elicited higher NT titers against the heterologous clade virus (A/whooper swan/Mongolia/244/2005 strain, clade 2.2) (Fig. 8C–D). The 83NNT HA mutant also shows the highest levels of neutralizing antibodies against H5pp of two heterologous clade viruses (A/Anhui/1/2005 strain, clade 2.3.4; A/Hubei/1/2010 strain, clade 2.3.2) (Fig. 9A–B). The results were obtained by two-dose priming with DNA-HA vector (hyperglycosylated HA mutants) and a third booster dose of FliC-VLPs that only contained the wild type HA. Using the FliC-VLPs containing only 83NNT and/or 127 NNS mutant HA antigen(s) as the third booster dose should further improve the elicited neutralizing antibody responses against HPAI H5N1 viruses.

The 83NNT mutation is located at the Cb antigenic site, and the 127NNS mutation is close to the Sa antigenic site of the HA of H5N1 human isolates, based on the H1N1 antigenic map [34], [35]. Glycan masking of these two sites may refocus polyclonal antibody responses to other antigenic sites of HA molecules. Recent studies have revealed that the cross-neutralizing epitopes of influenza viruses were mapped to either the F subdomain of HA2 stem [36], [37], or close to the receptor binding site [38], [39], the Ca2 site of H1N1 [38], as well as the A and B site/sites of H3N2 HA [40]. Since the HA1 globular head contains immunodominant epitopes that overlap with the extensive antigen variation sites compared to the non-overlapping of the poorly immunogenic HA2 stem region [41]. The mechanisms of glycan masking on 83NNT and 127NSS residues to enhance functional antibodies against homologous and heterologous clades of HPAI H5N1 viruses require further investigation.
